# Data annotation and its evaluation in artificial intelligence-based anatomy recognition for ultrasound-guided regional anesthesia: a clinical perspective

**DOI:** 10.3389/fmed.2026.1823745

**Published:** 2026-05-29

**Authors:** Bernard V. Delvaux, Yoann Elmaleh, Alwin Chuan, Alex T. Sia, Rajnish K. Gupta, Kristopher M. Schroeder, Karim Guessous, James S. Bowness

**Affiliations:** 1 Department of Anaesthesiology and Perioperative Medicine, Ramsay Santé, Private Hospital Claude Galien, Quincy-Sous-Sénart, France; 2 Department of Anaesthesia, Liverpool Hospital, Faculty of Medicine & Health, University of New South Wales Sydney, Sydney, NSW, Australia; 3 Department of Women's Anaesthesia, KK Women's and Children's Hospital, Singapore, Singapore; 4 Department of Anesthesiology, Vanderbilt University Medical Center, Nashville, TN, United States; 5 Department of Anesthesiology, University of Wisconsin School of Medicine and Public Health, Madison, WI, United States; 6 Department of Anaesthesia, University College Hospitals London, London, United Kingdom; 7 Department of Targeted Intervention, University College London, London, United Kingdom

**Keywords:** annotation, artificial intelligence, data quality, evaluation, segmentation, ultra-sound guided regional anesthesia

## Introduction

**Figure 1:**
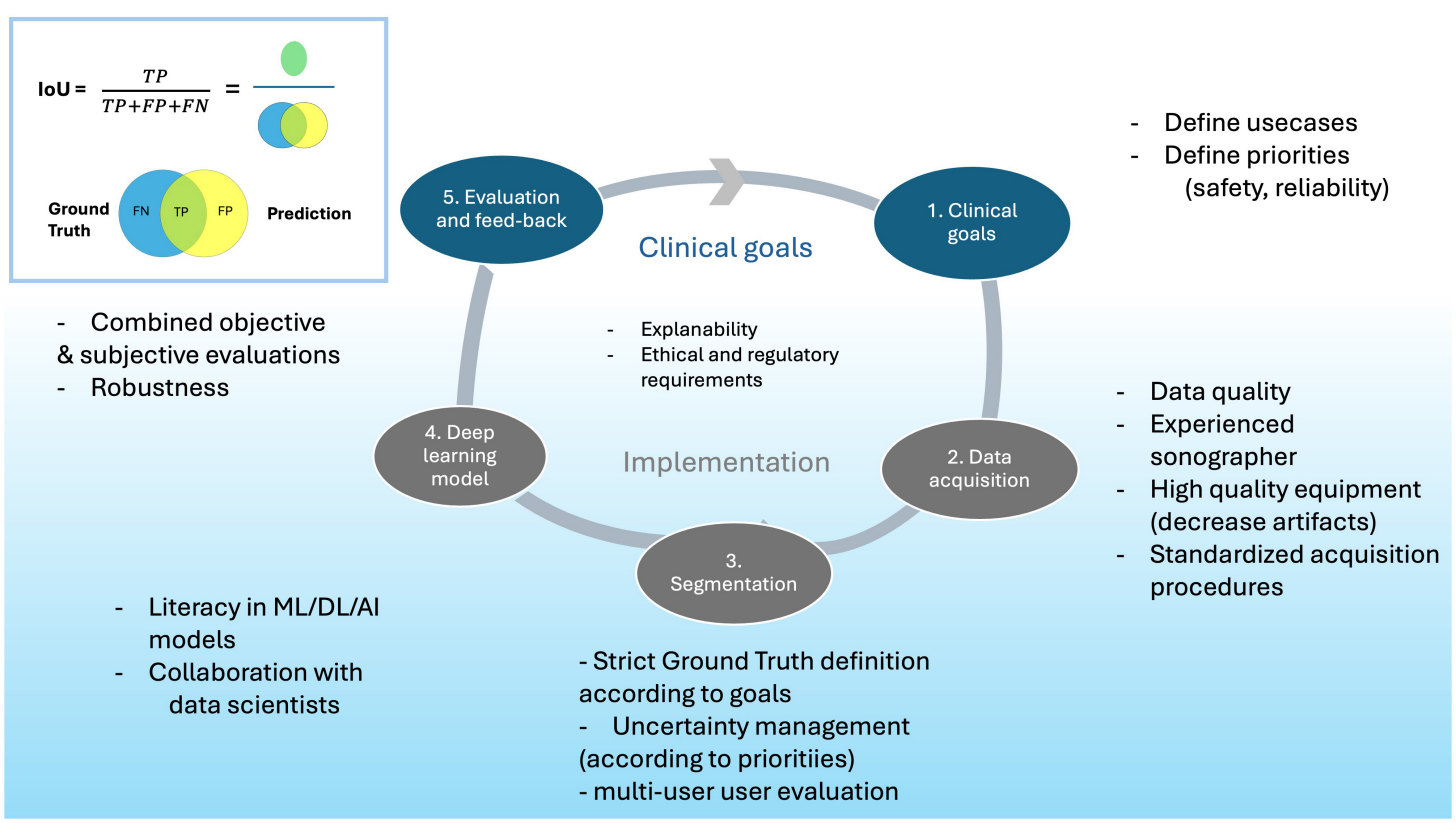
Overview of the involvement of clinicians at each step of the workflow. ML: machine learning; DL: deep learning; AI: artificial intelligence.

For many years, AI-based anatomy recognition in UGRA were evaluated primarily through objective technical metrics, because these were the only assessment tools available during early development. Such metrics quantify agreement between model outputs and a predefined reference, but do not define what makes them clinically useful. Furthermore, to our knowledge, the literature has poorly addressed the influence of clinical endpoints on their use. For instance, should the priority be to avoid missed anatomy or to limit unnecessary annotation? This issue has not been adequately considered at either the training or the evaluation stages. We have linked this insufficient standardization of training data and evaluation methods to substantial variability in performances (1–5). As these techniques were integrated into commercial ultrasound systems, it became possible to assess their usability alongside their technical performance. Notably, AI-assisted anatomy recognition in UGRA represents one of the most extensively implemented applications of AI in anaesthesia to date (6), even though important research gaps remain. In this opinion paper, we argue that this accumulated experience in metrics aligned with clinical anesthesiology and other medical fields could now inform better how such systems are trained, annotated, and evaluated (Figure 1).

## Data quality, annotation, and evaluation

### Clinical objectives and data acquisition

AI development should be guided by clinical needs (7, 8). Boosting novice confidence and serving as a teaching aid were initially described (9, 10), but new use cases may be documented, such as assisting experienced users in difficult-to-image patients with high BMI, oedema or amyotrophy (11). At this stage, safety and reliability priorities should also be considered since they will influence the annotation process (developed later). So far, current AI-based guidance for UGRA mainly improves procedural orientation but provides limited safety gains due to imperfect contour accuracy (2).

### Data

Data quality is a first prerequisite for robust model development. Images acquired by expert operators, using appropriate equipment (12) and standardised scanning protocols (13, 14), provide a reliable basis for annotation and training. At the same time, poor-quality images, although often unsuitable for accurate labeling, reflect real-world practice and therefore cannot simply be ignored. Increasing dataset size may partly mitigate this issue, but additional strategies are available. One approach is to annotate higher-quality images while training models on paired versions with artificially degraded quality, thereby exposing them to more realistic variability (15). Domain-adaptation methods may further reduce the mismatch between optimized training datasets and real-world clinical ultrasound, improving generalisability (16). Finally, deep-learning-based super-resolution and related enhancement techniques may help recover useful information from low-resolution or blurred frames (17, 18).

### Segmentation: the annotation of data

**Figure 2:**
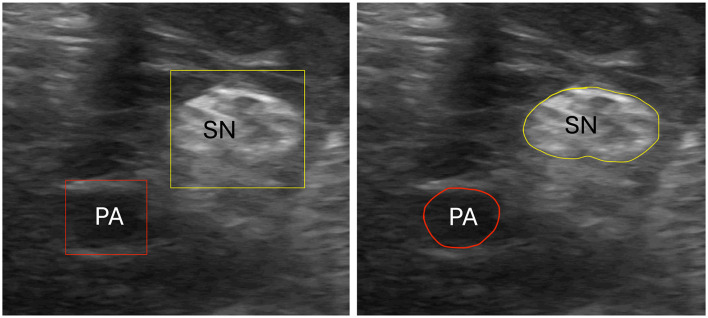
Two approaches of segmentation at the popliteal region. **Left**: object detection (bounding boxes); **right**: semantic segmentation (precise delineation). PA: popliteal artery (red); SN: sciatic nerve (yellow).

**Segmentation** is the process marking anatomical features that serve as a reference standard known as ‘ground truth'. The ground truth is then the anatomical region that is known (or assumed) to be true. It will be used to both train and evaluate the model. Two annotation strategies are usually used (Figure 2): semantic segmentation, which involves precise pixel-level delineation of anatomical structures (the most widely adopted approach in both research and commercial systems); and object detection by ‘bounding boxes', which involves the use of rectangular shapes to surround the region of interest.

Two aspects of segmentation will be considered: (1) the definition of precise segmentation rules and (2) the aggregation of annotations of several experts.

### Defining annotation rules

Robust criteria must be applied consistently to ground-truth annotation for both training and evaluation of the model, a standard that, to our knowledge, is not often reported in the current literature. For example, including paraneurium (or not) in the annotation of the nerve likely represents a significant source of variability (19). Furthermore heterogeneous “honeycomb” structure of the nerve or limited resolution and artifacts may blur margins of the anatomy. (4).

Uncertainty management should guide the the annotation strategy. Labeling only unequivocal regions favors precision (positive predictive value), potentially improving procedural safety by reducing misleading false-positive detections (although some nerves may be missed). Conversely, tolerating ambiguity favors recall (sensitivity) by highlighting plausible nerve locations, but may require greater expertise to select the relevant structure among multiple candidates. Whether precision or recall should be favored in model training remains elusive.

Automated or semi-automated methods (including thresholding, graph cuts, edge-based models, deformable models, deformable image registration) may be helpful in this process. These techniques are computer-assisted techniques that help outline the region of interest, using differences in density and texture, edges, expected anatomical shape, and comparisons with anatomical atlases (20). More simply, simple interpolation between segmented slices can be performed in most segmentation softwares (21). Furthermore, self-supervised and unsupervised techniques, that allow better segmentation with far less annotations requirements. *Self-supervised learning* uses huge amounts of unannotated data to find useful patterns (22, 23). *Contrastive learning* teaches the model to bring similar images (like the same structure from different angles) closer and push different ones apart. *Unsupervised clustering* can group pixels or regions into meaningful classes without labels.

### Aggregation of segmentation of different experts

The literature has advocated combining multiple readers' opinions to reduce the flaws associated with a single evaluator's annotation, including subjective perception, even among experts (24). Several approaches are available.

Firstly, ‘label fusion' approaches make a consensual choice among experts. For example, ‘majority vote' gives equal weight to all votes, and Simultaneous Truth and Performance Level Estimation (STAPLE) gives weights according to the experience of each annotator (25, 26). These approaches are standard practice in radiation oncology and have even led to the creation of segmentation atlases (27, 28). An alternative approach is adjudication (also termed arbitration or consensus), in which readers jointly resolve disagreements has been proposed as a high-quality reference standard for image interpretation (29). Even though examples can be found in UGRA-based annotations, there is, again, no consensus in this field (30, 31).

Secondly, direct multi-rater training considers both agreement and disagreement and assigns probabilities to each. These methods are based on probabilistic or machine/deep learning models and reflect the ambiguity/variation related to different human annotations by modeling a ‘latent' truth (a truth that may be hidden but is identifiable) (32, 33).

Thirdly, segmentation distribution models based on deep learning (for example Probabilistic U-Net) reflects the intrinsic ambiguity of the data (no strict truth can be identified, but there is a distribution of probability), which is particularly useful when the data are not precise enough to find a certain latent truth (34).

In summary, uncertainty management - whether labels should cover only unequivocal pixels or also plausible regions, and multi-rater strategies - is set upstream by annotation policy. Prior work shows that these design choices propagate to model behavior and evaluation (35). Practical implementations include logging per-label confidence scores and, where appropriate, using uncertainty-aware supervision such as confidence-weighted masks or confidence boxes. Despite their promise, such uncertainty-aware practices are not currently implemented in ultrasound image annotation workflows for regional anesthesia.

### Model training

Even though model training is beyond the scope of this article, we think it is essential that clinicians interact with data scientists who manipulate those models and become familiar with some of their characteristics. For instance, the distinction between frame-by-frame analysis (every image is analyzed without taking previous images into account – used in most current models) or ‘tracking' analysis (where previous images are taken into account for analysing the present image) should be known.

We think literacy in AI among doctors should also be promoted (e.g. by mandatory workshops on model bias and performance metrics, joint journal clubs with data scientists, hands-on sessions, and multidisciplinary review of model errors). Pitfalls are pervasive [e.g. overconfidence bias (36)]. Integrating principles of explainable AI (XAI) (37) may be valuable strategies to understand involved heuristics (e.g. saliency maps (38) - highlighting relevant features in the ultrasound image - combined to uncertainty maps). Beyond technical implementation, models implementation should also be aligned with applicable regulatory pathways (including FDA and CE marking), as several factors (including risk classification and clinical claims) will shape the evidence required for deployment.

### Evaluating performance and clinical utility

The commercial implementation of AI-driven anatomy recognition models on ultrasound machines makes evaluating those systems, including their usability, much more feasible on a large scale. The segmentation can be evaluated using either objective or subjective metrics - two complementary approaches. (2, 3, 39).

**Objective metrics** were almost exclusively used before commercial implementation. They provide a standardized means of a pixel-level evaluation (every pixel is classified as true positive, false positive and false negative), that quantify overlap between the model prediction and the reference standard. Commonly used metrics include the Dice Similarity Coefficient (DSC), Intersection over Union (IoU), precision (positive predictive value), recall (sensitivity), specificity, accuracy, and the Hausdorff distance, though this list is not exhaustive (40) (Supplementary Figure). For example, IoU and DSC range from 0 (no overlap) to 1 (perfect overlap). These metrics offer precise and reproducible quantification. However, they have important limitations. First, despite occasional proposals (e.g., IoU ≥ 0.5), there is no consensus on acceptable thresholds, so comparisons are often made against prior results in related domains. Second, objective scores may diverge from expert evaluation and clinical utility: models can be judged helpful by clinicians while achieving only modest numerical performance (3, 11). Subjective metrics are then desirable to give clinical meaning to those objective metrics.

Expert subjective evaluation (41) relies on predefined quality criteria, including assessment of overall highlighting quality, clinical utility, and operator confidence during the procedure. However, it remains unclear which specific criteria should be prioritized in different clinical situations. Several rating instruments have been proposed; for example, Lloyd et al. described a four-item scale assessing (i) overall relevance of the highlighting for UGRA, (ii) segmentation quality, (iii) utility for identifying the region of interest, and (iv) anticipated usefulness for less experienced operators (42). However, to our knowledge, validation - using a formal workflow including testing validity and reliability (43) - is still needed in the field of AI-based anatomy recognition for UGRA.

Objective and subjective approaches have to combine in future efforts to validate solid quantitative or semi-quantitative scales of clinical use. Defining thresholds for those metrics depends on clinically relevant decisions. For example, the quality of segmentation necessary to orientate the procedure for an expert and novice is probably very different, making subjective evaluation a necessary supplementary step.

Multi-evaluator consensus methods that have been outlined for segmentation purposes may also be employed for evaluation, thereby enhancing the robustness of the process. However, to the best of our knowledge, few studies have applied this promising but time-consuming method in UGRA (44).

Another aspect of evaluation is robustness assessment. Robustness is the ability of a model to maintain a consistent and reliable level of performance, even when faced with unexpected data, noise or unexpected situations. It has different aspects and measures (45). One notable example includes the use of "edge cases”, where borderline, atypical or challenging patient scenarios are presented to the model so see how it reacts. Examples of such cases include patients with complex anatomical variations, diverse body habitus, or significant pathological alterations. Additionally, adversarial testing (introducing visually negligible pixel-level perturbations, such as changes in speckle or frequency content) provides insights into the model's resilience. Finally the evaluation should not only consider the performance of the system, but also its explainability (37).

## Discussion

The usefulness of AI for UGRA depends on the quality of the training data and segmentation, and on how rigorously the latter is evaluated in relation to clinical utility. Clinicians are involved in all steps of the workflow (Figure 1), and their domain knowledge should inform how these steps are implemented. This paper emphasizes that progress requires standardizing what constitutes “ground truth” and how uncertainty is managed. To address inter-observer variability, consistent annotation rules, a systematic use of multi-rater strategies (from simple fusion to probabilistic truth models).

Regarding evaluation, objective measures should be reported at pre-specified operating points that reflect the intended clinical task. Further work is needed to determine which aspects of performance matter most in different clinical settings, ideally through structured expert consensus processes, and to develop and validate rating instruments. Finally, robustness testing (including adversarial and “edge-case” challenges) and assessment of explainability should be integrated into evaluation frameworks.

This opinion paper has important limitations. Several concepts are extrapolated from other areas of artificial intelligence and medical imaging rather than supported by UGRA-specific validation studies. The fact that UGRA has distinctive features may limit direct translation from other imaging domains. Accordingly, the proposed framework should be considered hypothesis-generating, rather than formal UGRA-specific recommendations. Further studies are needed to adresses the transferability to the proposed concepts.
