# Supplementary figures and images for: Data annotation and its evaluation in artificial intelligence-based anatomy recognition for ultrasound-guided regional anesthesia: a clinical perspective
Source: Front Med (Lausanne). 2026 May 29;13:1823745. doi: 10.3389/fmed.2026.1823745 (PMC13260577; doi:10.3389/fmed.2026.1823745)

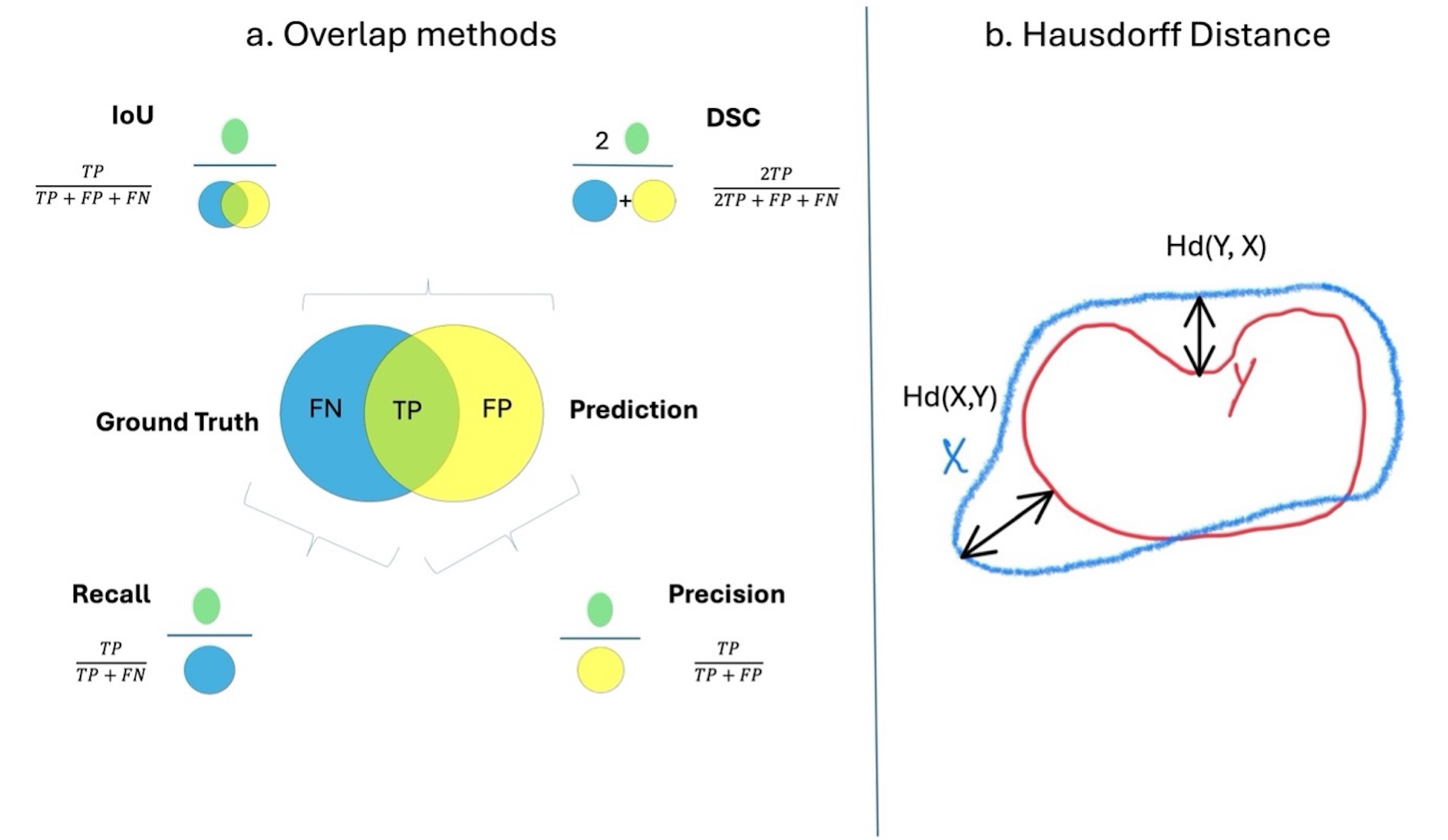

Supplement: Supplementary file 1 [file Figure_1.jpg]
